# PHENOTYPIC ANTIBIOTIC RESISTANCE PROFILES OF GRAM-NEGATIVE BACTERIA IN CLINICAL SAMPLES PRE AND DURING COVID-19 PANDEMIC AT ZONAL REFERRAL HOSPITAL NORTHERN TANZANIA

**DOI:** 10.21010/Ajidv19i2S.1

**Published:** 2025-10-17

**Authors:** VUAI Miza Silima, KUMBURU Happiness Houka, MKUMBAYE Sixbert Isdory, KAJEGUKA Debora Charles, KAVISHE Reginald Adolph

**Affiliations:** 1 Department of Biochemistry and Molecular Biology, KCMC University, Moshi, Tanzania; 2 Department of Microbiology and Parasitology, The State University of Zanzibar, Moshi, Tanzania; 3 Department of Microbiology and Immunology, KCMC University, Moshi, Tanzania; 4 Department of Biotechnology, Kilimanjaro Clinical Research Institute, Moshi, Tanzania; 5 Department of clinical laboratory, Kilimanjaro Christian Medical Centre, Moshi, Tanzania

**Keywords:** Antimicrobial, resistance, COVID-19, and Gram- negative bacteria

## Abstract

**Background:**

COVID-19 has aggravated antimicrobial use owing to limited treatment options, raising concerns about antimicrobial resistance, which was previously estimated to potentially cause 10 million global deaths within 30 years. This study evaluated the potential impact of the COVID-19 pandemic on antibiotic resistance in referral hospitals.

**Material and Methods:**

A cross-sectional study at Kilimanjaro Christian Medical Centre analyzed clinical bacterial samples from pre-COVID-19 (2018) and during COVID-19 (2020, 2023). Two hundred isolates from 2018 and 2020, and 121 samples from 2023, were examined. Bacterial isolates were identified using API 20E for Enterobacteriaceae (bioMérieux) and standard biochemical tests, while antimicrobial susceptibility was assessed using the disc diffusion method.

**Results:**

During the COVID-19 pandemic, antibiotic resistance among bacteria has increased significantly. Resistance to ampicillin 113 (95.8%, p=0.018), ceftriaxone 102 (74.5%, p=0.043), and ciprofloxacin 119 (68.8%, p=0.003) increased. Conversely, resistance to chloramphenicol 19 (16.1%, p=0.021) and amoxicillin-clavulanic acid decreased to 62 (52.5 %, p = 0.007). *Klebsiella pneumoniae* showed decreased resistance to chloramphenicol 11(20.8%, p=0.004) and amoxicillin-clavulanic acid 27(50.9%, p=0.034). *Acinetobacter species* also showed a significant increase in ceftriaxone resistance 18(94.7%, p=0.018). Among the 233 isolates, ESBL-producing bacteria were identified in 101 (43.4%), and *Klebsiella pneumoniae* and *Escherichia coli* showed the highest frequencies at 40 (39.6%) and 54 (53.5%), respectively.

**Conclusion:**

During COVID-19, ampicillin, ciprofloxacin, and ceftriaxone resistance significantly increased, whereas restricted antibiotics, such as meropenem, showed lower resistance. The extensive and uncontrolled use of antibiotics during the pandemic has aggravated antimicrobial resistance, necessitating intensified and coordinated efforts to combat it.

## Introduction

Antibiotic resistance has led to bacterial infections becoming one of the foremost causes of disturbing health effects, posing a significant threat to public health (Nwobodo *et al.*, 2022). Several studies have reported effects of antibiotic resistance on clinical and therapeutic outcomes, such as higher rates of morbidity and death, longer hospital stays, increased healthcare costs, and the need for more expensive alternative medications (Nwobodo *et al.*, 2022). In 2019, antimicrobial resistance (AMR) was estimated to have caused 1.27 million deaths globally. Sub-Saharan Africa experienced the highest impact, with 27.3 deaths per 100,000 directly attributed to AMR (where AMR was the primary cause of death) and 114.8 deaths per 100,000 associated with AMR (including cases where AMR contributed to, but was not necessarily the sole cause of death)(Murray *et al.*, 2022). According to the WHO, in the African region in 2019, approximately 1.05 million fatalities were linked to AMR, while 250,000 deaths were specifically attributed to AMR (Sartorius *et al.*, 2023).

In 2024, the WHO updated a list of antibiotic-resistant priority bacteria that pose a serious risk to humans, for which new antibiotics are urgently needed and strategies to prevent and mitigate the spread of AMR. The list is ranked based on the urgency of the need for new antibiotics, with critically important bacteria, including *Acinetobacter baumannii* and *Enterobacteriaceae* resistant to carbapenems and beta-lactams. *Pseudomonas aeruginosa* is classified as a high priority because it is resistant to last-line antibiotics, highly transmissible, particularly in healthcare settings, and has high mortality rates (OMS, 2024).

During pre-COVID-19 analysis, it is estimated that AMR will lead to approximately 10 million deaths globally in the next 30 years, outcompeting mortality rates from cancer (8.2 million), diabetes (1.5 million), and other causes(Rizvi and Ahammad, 2022). The COVID-19 pandemic has disrupted antibiotic stewardship efforts and has significantly increased the worldwide consumption of antibiotics and the use of local traditional medicines without fully understanding the dangers and biocides worsening the ongoing global AMR crisis (Rizvi and Ahammad, 2022; Djuikoue *et al.*, 2023). A study conducted in Egypt, Iraq, Lebanon, and Jordan found a significant increase in AMR during the COVID-19 outbreak (Bizri *et al.*, 2023). This increase was linked to several reasons; including increased hospital occupancy, a shift in attention away from AMR monitoring, changes in AMR patterns, and an increase in unnecessary and unjustified antibiotic prescriptions during the pandemic (Bizri *et al.*, 2023). Furthermore, Sulayyim *et al*. (2022), in a systematic review of 23 studies, demonstrated an increase in antibiotic resistance during the COVID-19 pandemic. The most commonly reported resistant gram-negative bacteria are *Acinetobacter baumannii*, followed by *Klebsiella pneumoniae*, *Escherichia coli*, and *Pseudomonas aeruginosa* (Sulayyim *et al.*, 2022). High resistance rates against gram-negative bacteria have been reported in Tanzania (Neema *et al.*, 2023). Nevertheless, a notable gap remains in understanding how the emergence of the COVID-19 pandemic has influenced antibiotic resistance patterns in Tanzania. Tanzania is among the few countries that did not institute strict lock-downs, and interactions between individuals and communities continued during the pandemic with minimum social distancing, hand hygiene, avoiding unnecessary gatherings, and limiting attendance at funerals as the main measures taken (Hamisi *et al.*, 2023). This study aimed to elucidate the potential impact of the COVID-19 pandemic on antibiotic resistance in referral hospitals.

## Material and Methods

### Study design and setting

This descriptive cross-sectional study included two distinct periods: the pre-COVID-19 pandemic period in 2018, and the COVID-19 period from 2020 to 2023. The study was conducted at the Kilimanjaro Christian Medical Center (KCMC), a zonal referral hospital located in Northern Tanzania with a bed capacity of 630, serving a population of over 15 million people.

### Sample collection

Purposive sampling was used to collect 121 clinical samples from the inpatients admitted to the medical and surgical wards at KCMC in 2023. The clinical samples included 37 urine samples, 27 blood samples, and 57 wound swabs/pus samples collected from March to June 2023. Furthermore, 200 bacterial isolates from blood (86), cerebrospinal fluid (40), sputum (16), urine (1), and wound swabs/pus (63) were obtained from the archives at the Kilimanjaro Clinical Research Institute (KCRI) biorepository in 2018 and 2020.

Bacterial isolates were collected from clinical samples during both periods from two projects: Whole Genome Sequencing (WGS) and DUKE-EMECaD, and archived at KCRI. These samples were stored at -80^0^C in the KCRI biorepository. WGS samples were obtained from inpatients admitted to KCMC with the objective of characterizing infectious bacteria using whole genome sequencing. DUKE-EMECaD samples were collected from patients who died shortly after hospital admission at KCMC, with the aim of identifying knowledge gaps regarding the actual pathological, evidence-based cause of death among patients with short-term hospitalization.

### Bacteria Culture and Identification

Urine samples were cultured on CLED agar (Oxoid, United Kingdom) and blood agar (Himedia, India), whereas wound or pus swab samples were cultured on MacConkey agar and blood agar (Himedia, India) and incubated at 37 °C for 24 h. Blood samples were incubated in the BACTE system for a maximum of five days. Positive blood cultures were then cultured on MacConkey agar (Himedia, India) and blood agar (Oxoid, United Kingdom), and incubated for 24 h at 37 ^0^C with 5% CO_2_. Bacterial isolates from the KCRI biorepository were cultured on blood agar (Oxoid, United Kingdom) to check for purity, and those that had mixed colonies were subcultured. Gram staining was used to identify gram-negative and gram-positive bacteria. The bacterial isolates were identified using API 20E (bioMerieux, France) biochemical tests for *Enterobacteriaceae* and conventional biochemical tests including triple sugar iron, sulfur indole motility, citrate, and urease. The identified isolates were stored at -80^0^C.

### Drug susceptibility test

Antimicrobial susceptibility testing (AST) was performed using disc diffusion on Muller–Hinton Agar (Oxoid, United Kingdom), according to the Clinical Laboratory Standards Institute CLSI 2021 guidelines (CLSI 2021). This method allows for rapid and reliable determination of antibiotic susceptibility, and is widely used for routine microbiology laboratories, especially in resource-limited settings. Gram-negative bacteria were tested for chloramphenicol (30µg), ceftriaxone (30µg), gentamicin (10µg), ciprofloxacin (5µg), ampicillin (10µg), amikacin (30µg), ceftazidime (30µg), amoxicillin-clavulanic acid (30µg), cefoxitin (30µg), trimethoprim-sulfamethoxazole (23.75 µg / 1.25 µg), meropenem (10 µg), tetracycline (30µg), and piperacillin-tazobactam (100/10µg). Interpretation as susceptible, intermediate, or resistant was performed according to CLSI 2021. Isolates with intermediate or resistant results were merged into a single resistant group during data analysis. Considering the intermediate group, resistance can be one way to avoid uncertain therapeutic effects (Kumburu *et al.*, 2017). *Escherichia coli* ATCC 25922 was used for quality control.

### Phenotypic Screening and confirmation for Extended-spectrum beta-lactamase (ESBL) producing isolate

The ESBL screening test was performed using the standard disk diffusion method with two antibiotic discs ceftazidime (30μg), and ceftriaxone (30μg) (Oxoid, United Kingdom). These antibiotic discs were placed on Muller-Hinton agar (and incubated at 37°C for 18–24 h. The breakpoints for suspicion of ESBL production were ≤ 25 mm for ceftriaxone (30 g) and ≤ 22 mm for ceftazidime (30 g) (Salihu *et al.*,2021; Teferi *et al.*, 2023).

ESBL was confirmed using the double-disk synergy (DDS) method. A susceptibility disk containing amoxicillin/clavulanate (30μg) was placed at the center of the plate. Two disks of cephalosporin agents, ceftazidime (30μg) and ceftriaxone (30μg), were located 15 mm away from the center of the amoxicillin/clavulanate disk. All the cultured plates were incubated aerobically overnight at 37°C. A positive result was defined as a ≥5 mm increase in the zone of inhibition diameter for each cephalosporin-clavulanate disk combination in comparison to the zone diameter of the corresponding cephalosporin disk, which was interpreted as an ESBL producer. *Escherichia coli* ATCC 25922 served as a negative control (Salihu *et al.*, 2021; Sageerabanoo *et al.*, 2015).

### Ethical clearance

Ethical clearance was obtained from the KCMC University, Research Ethical Review Committee with clearance certificate number: No, 2601.

### Data Analysis

Data were entered in an Excel sheet and exported to STATA 15 (StataCorp, Texas, USA). Descriptive analysis wasotypic antibiotic resistance patterns were tested using th summarized as frequencies and percentages, and differences in the phene chi-square (χ2) test. Statistical significance was set at p ≤ 0.05.

## Results

Of the 121 freshly collected samples, 95 showed positive growth, and 26 showed negative growth. Among the 95 positive samples, 9 were identified as gram-positive cocci, 1 as Bacillus spp., and 85 as gram-negative bacteria. Of the 85 samples, 118 Gram-negative bacterial isolates were obtained, including 34 (28.8%) *Escherichia coli*, 23 (19.5) *Klebsiella pneumoniae*, 20 (16.9%) *Pseudomonas spp*., 17 (14.4%) *Acinetobacter spp*., 8 (0.07%) *Proteus spp*., 6 (0.05%) *Enterobacter spp*., 5 (0.04%) *Morganella morganii*, 1 (0.01%) *Pantoea spp*., 3 (0.03%) *Citrobacter freundii*, and 1 (0.01%) *Raultella ornithinolytica*. The focus was narrowed to *Escherichia coli*, *Klebsiella pneumoniae*, *Pseudomonas spp*., and *Acinetobacter spp*., totaling 94 bacterial isolates for further investigation. Additionally, 200 bacterial isolates were obtained from the KCRI biorepository, of which 58 were unable to grow and 142 were able to grow, resulting in 63 isolates in 2018 and 79 in 2020. Combining 94 freshly collected isolates with 142 revived archived isolates yielded 236 gram-negative bacterial isolates for further study. Of these, 63 (26.7%) were isolated before COVID-19 and 173 (73.3%) were isolated during the COVID-19 era. The majority were *Escherichia coli* 95 (40.3%), followed by *Klebsiella pneumoniae* 62 (26.3*%), Pseudomonas spp* 53 (22.5%), and *Acinetobacter spp* 26 (11%). These isolates originated from various clinical specimens, including 27(11.4%) from urine, 102(43.2%) from wound swabs/pus, 22 (9.3%) from cerebrospinal fluid (CSF), 73 (30.9%) from blood, and 12 (5.1%) from sputum. *Escherichia coli* predominated in blood 37(50.7%), urine 12(44.4%), and wound samples 35(34.3%); *Klebsiella pneumoniae* was predominantly found in CSF 10 (45.5%) and blood 23 (31.5%) (Table 1b).

**Table 1:** Distribution of bacteria isolates among different specimens

Specimens type						

Organisms	Blood	CSF	Sputum	urine	wound swab/pus	Total
*Acinetobacter spp.*	3(4.1%)	3(13.6%)	2(16.7%)	4(14.8%)	14(13.7%)	26
*Escherichia coli*	37(50.7%)	4(18.2%)	7(58.3%)	12(44.4%)	35(34.3%)	95
*Klebsiella pneumoniae*	23(31.5%)	10(45.5%)	2(16.7%)	5(18.5%)	22(21.6%)	62
*Pseudomonas spp.*	10(13.7%)	5(22.7%)	1(8.3%)	6(22.2%)	31(30.4%)	53

Total	73	22	12	27	102	236

### Overall antibiotics resistance rate of bacteria isolates in both study periods

**Figure 1:**
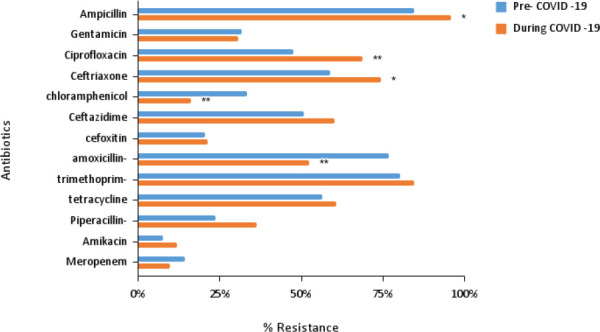
Overall antibiotic resistance rate in both study periods, before and During the COVID 19 era; * indicates p ≤ 0.05, ** indicates p ≤ 0.01.

The bacteria isolated in this study had various resistance rates. Across both periods under study. The resistance rates were markedly high for several antibiotics: ampicillin, 33 (84.9%) to 113 (95.7%); trimethoprim-sulfamethoxazole, 37 (80.4%) to 116 (85.4%); amoxicillin/clavulanic acid, 30 (76.9%) to 62 (52.5%); ceftriaxone, 27(58.7%) to 102(74.5%); tetracycline, 26(56.5%) to 83(60.6%); ceftazidime, 32(50.8%) to 104(60.1%); and ciprofloxacin, 30 (47.6%) to 119 (68.8%). Lower resistance rates were observed for amikacin 3 (7.7 %) to 14 (11.9 %)), meropenem (9 (14.3 %) to 17 (9.8 %)), piperacillin-tazobactam 15 (23.8 %) to 63 (36.4 %)), cefoxitin (8 (20.5 %) to 25 (21.2 %)), chloramphenicol 13 (33.3 %) to 19 (16.1 %)) and gentamicin 20 (31.7 %) to 53 (30.6 %)). The resistance rates during the COVID-19 era showed statistically significant increases for ampicillin 113 (95.8%) (p = 0.018), ciprofloxacin 119 (68.8%) (p = 0.003), and ceftriaxone 102 (74.5%) (p = 0.043). A notable decrease in resistance rates was observed for Chloramphenicol 19 (16.1%) (p = 0.021) and Amoxicillin-clavulanic acid 62 (52.5%) (p = 0.007) (Figure 1).

### Antibiotics resistance Pattern of gram-negative bacteria before and During COVID 19 era

The antimicrobial resistance rates of *Escherichia coli*, *Klebsiella pneumoniae*, *Pseudomonas spp*., and *Acinetobacter spp*. were recorded. *Escherichia coli* isolates showed variable resistance rates against the tested antimicrobials in both study periods, with the highest resistance rates against trimethoprim-sulfamethoxazole 25 (83.3%), ampicillin 24 (80.0%), amoxicillin/clavulanic acid 22 (73.3%), and tetracycline 21 (70.0%) in the pre-COVID-19 period and against trimethoprim-sulfamethoxazole 60(93.9%), ceftriaxone 47(72.3%), ciprofloxacin 46 (70.8%), ceftazidime 43 (66.2%) during the COVID-19 period. Low resistance rates, ranging from 3.1% to 21.5%, were recorded against meropenem, amikacin, cefoxitin, and piperacillin-tazobactam during both study periods.

*Klebsiella pneumoniae* showed high resistance to trimethoprim-sulfamethoxazole 8 (88.9%) and amoxicillin/clavulanic acid 8 (88.9%) in the pre-COVID-19 and against ciprofloxacin 5 (81.1%), trimethoprim-sulfamethoxazole 42 (79.3%) and tetracycline 29 (74.7%) during COVID-19 period. Low resistance rates of *Klebsiella pneumoniae* were observed against amikacin and meropenem, ranging from 3.8% to 16.9% during both study periods*. Klebsiella pneumoniae* showed a statistically significant decrease in resistance rate against chloramphenicol 11(20.8%), p=0.004 and amoxicillin-clavulanic acid 27 (50.9%), p=0.034.

In the case of *Acinetobacter spp*., in the pre-COVID-19 period, the highest resistance rates were observed against ceftriaxone 4 (57.1%) and trimethoprim-sulfamethoxazole 4 (57.1%), and the lowest resistance rate was observed against meropenem 0 (0%). During the COVID-19 period, the highest resistance rates of *Acinetobacter spp*. were against ceftriaxone 18 (94.7%), ceftazidime 14 (73.7%), and piperacillin-tazobactam 15 (78.9%), whereas the lowest resistance rates were against tetracycline 5 (26.3%) and meropenem 7 (36.8%). *Acinetobacter spp*. showed a statistically significant increase in resistance rates against ceftriaxone during the COVID-19 period against ceftriaxone18 (94.7%) (p = 0.018).

*Pseudomonas spp*. showed highest resistance rate against ceftazidime 8 (47.1%), while the lowest resistance was noted against gentamicin and ciprofloxacin 2 (23.5%). During the COVID-19 period, increased resistance rates were observed for ciprofloxacin 17 (47.2%) and piperacillin-tazobactam 15 (41.8%), whereas a decreased resistance rate was noted for gentamicin 5 (13.9%), ceftazidime 10 (27.8%), and meropenem 6 (16.7%). *Pseudomonas spp*. did not show a statistically significant increase or decrease in the resistance rates between the study periods (Table 2).

**Table 2:** Antimicrobial resistance Pattern (R*) of isolated gram-negative bacteria

	*Escherichia coli*			*Klebsiella pneumoniae*			*Acinetobacter spp*			*Pseudomonas spp*		
Antibiotics	Pre COVID	During COVID	P-value	Pre COVID	During COVID	P-value	Pre COVID	During COVID	P-value	Pre COVID	During COVID	P-value
	R (%) (N=30)	R (%) (N=65)		R (%) (N=9)	R (%) (N=53)		R (%) (N=7)	R (%) (N=19)		R (%) (N=17)	R (%) (N=36)	
Ampicillin	24(80.0)	60(92.3)	0.081	9(100)	53(100)	-	NA	NA	-	NA	NA	
												
Gentamicin	9(30.0)	16(24.3)	0.58	5(55.6)	22(41.5)	0.432	2(28.6)	10(52.3)	0.275	4(23.5)	5(13.9)	0.383
												
Ciprofloxacin	19(63.3)	46(70.8)	0.469	5(55.6)	43(81.1)	0.09	2(28.6)	13(68.4)	0.068	4(23.5)	17(47.2)	0.1
												
Ceftriaxone	17(56.7)	47(72.3)	0.131	6(66.7)	37(66.8)	0.85	4(57.1)	18(94.7)	0.018*			
												
Chloramphenicol	7(23.3)	8(12.3)	0.171	6(66.7)	11(20.8)	0.004*	NA	NA	-	NA	NA	
												
Ceftazidime	15(50.0)	43(66.2)	0.133	6(66.7)	37(66.8)	0.85	3(42.9)	14(73.7)	0.143	8(47.1)	10(27.8)	0.167
												
Cefoxitin	6(20.0)	7(10.8)	0.224	2(22.2)	18(33.9)	0.486	NA	NA	-	NA	NA	
												
Amoxicillin- clavulanic acid	22(73.3)	35(53.9)	0.072	8(88.9)	27(50.9)	0.034*	NA	NA	-	NA	NA	
												
Trimethoprim-sulfamethoxazole	25(83.3)	61(93.9)	0.104	8(88.9)	42(79.3)	0.498	4(57.1)	13(68.4)	0.592	NA	NA	
												
Tetracycline	21(70.0)	49(75.4)	0.58	3(33.3)	29(74.7)	0.235	2(28.8)	5(26.3)	0.908	NA	NA	
												
Piperacillin-tazobactam	4(13.3)	14(21.5)	0.343	3(33.3)	9(35.9)	0.884	3(42.9)	15(78.9)	0.077	5(29.4)	15(41.8)	0.39
												
Amikacin	3(10.0)	5(7.7)	0.707	0(0)	9(16.9)	0.181	-	-		-	-	
												
Meropenem	3(10.0)	2(3.08)	0.16	1(11.1)	2(3.8)	0.343	0(0)	7(36.8)	0.06	5(29.4)	6(16.7)	0.286

N: number of cases tested against the antibiotics; R%: percentage of antimicrobial resistance;

* statistically significant change; -: not tested.

### Trend of antibiotics resistance among bacteria isolates

Significant changes in bacterial resistance to various antibiotics have been noted between 2018, 2020, and 2023. *Escherichia coli* consistently showed high resistance to trimethoprim-sulfamethoxazole throughout these years. Resistance to Ampicillin was also notably high, with rates of 24 (80.8%) in 2018, 27 (87.1%) in 2020, and 33 (97.1%) in 2023. Similarly, resistance to ciprofloxacin remains high, with rates of 19 (63.3%) in 2018, 20 (64.5%) in 2020, and 29 (76.5%) in 2023. A significant change in resistance to ceftriaxone was observed, from 17 (56.7%) in 2018 to 29 (85.3%) in 2023 (p=0.021). Resistance to Ceftazidime is high, with figures of 15 (50%) in 2018,18(58.1%) in 2020, and 25 (73.5%) in 2023. In contrast, resistance to cefoxitin was low at 6 (20%) in 2018, 1 (3.1%) in 2020, and 6 (18%) in 2023. Chloramphenicol resistance was low 7 (23.3%) in 2018, 4(12.8%) in 2020 and 4 (11.8%) in 2023. Resistance to amoxicillin-clavulanic acid remained high, exceeding 50% across all three years. *Escherichia coli* demonstrated consistently low resistance to gentamicin at rates of 9 (30%) in 2018, 4 (12.9%) in 2020, and 12 (35.3%) in 2023. Resistance to Piperacillin-tazobactam was low, ranging from 9 (13.3%) in 2018 to 5 (16.1%) in 2020 and 9 (26.5%) in 2023. Meropenem resistance was generally low in *Escherichia coli*, with rates of 3 (10%) in 2018, compared to 1(3.2%) in 2020 and 2023. Lastly, *Escherichia coli* showed low resistance to amikacin at levels of 3 (10%) in 2018,1 (3.2%) in 2020, and 4 (11.8%) in 2023 (Figure 2A).

**Figure 2A:**
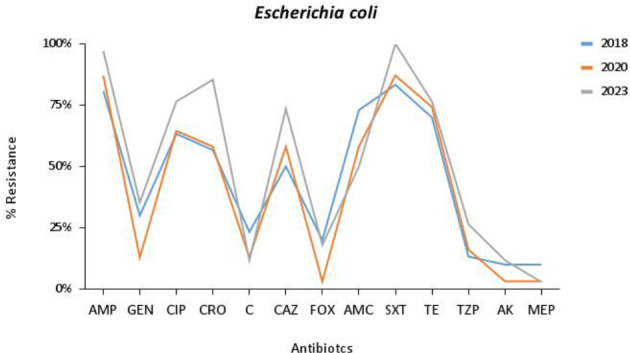
Trends in antimicrobial resistance of *Escherichia coli* in 2018, 2020, and 2023. AMP =Ampicillin, GEN= Gentamicin, CIP= ciprofloxacin, TZP= piperacillin-tazobactam, MEP= meropenem, CAZ = ceftazidime, CRO=ceftriaxone, SXT= trimethoprim-sulfamethoxazole, AK= amikacin, TE= tetracycline, C =Chloramphenicol, FOX=cefoxitin, AMC= amoxicillin clavulanic acid. *Escherichia coli* showed statistically difference in resistance to CRO, p=0.021.

*Klebsiella pneumoniae* showed significant changes in resistance rates to trimethoprim-sulfamethoxazole, with resistance recorded at 8 (88.9%) in 2018, 20 (66.7%) in 2020, and 23 (100%) in 2023 (p=0.006). Resistance to ampicillin remained consistently high at 100% across all three years. Ciprofloxacin resistance was high at 19 (82.6%) in 2023 and 24 (80%) in 2020, compared to 5 (55.6%) in 2018. Resistance to ceftazidime remains high, with rates of 6 (66.7%) in 2018, 18 (60.0%) in 2020, and 19 (82.6%) in 2023. Cefoxitin resistance varied; it was low at 2 (22.2%) in 2018, high at 12 (40.0%) in 2020, and then low at 6 (26.1%) in 2023. Additionally, resistance to chloramphenicol significantly varied, reaching 6 (66.7%) in 2018, and low to7(23.3%) in 2020, and 4 (17.4%) in 2023 (p=0.015). Resistance to amoxicillin-clavulanic acid showed high variability in 2018, with a rate of 8 (88.9%), low in 2020 with a rate of 12 (40%), and high to 15 (65.2%) in 2023 (p=0.02). Gentamicin resistance was high in 5 (55.6%) patients in 2018 but low in 11 (36.7%) in 2020 and 11 (47.8%) in 2023. Resistance to Piperacillin-tazobactam remains below 50% across all years, recorded at 3 (33.3%) in 2018, 13 (43.3%) in 2020, and 9 (26.1%) in 2023. Meropenem resistance was consistently low, with rates of 1 (11.1%) in 2018 and similarly low at 1 (3.3%) and 1 (4.4%) in 2020 and 2023, respectively. Lastly, amikacin resistance was low 0(0%) in 2018, 4 (13.3%) in 2020, and 5 (21.7%) in 2023 (Figure 2B).

**Figure 2B:**
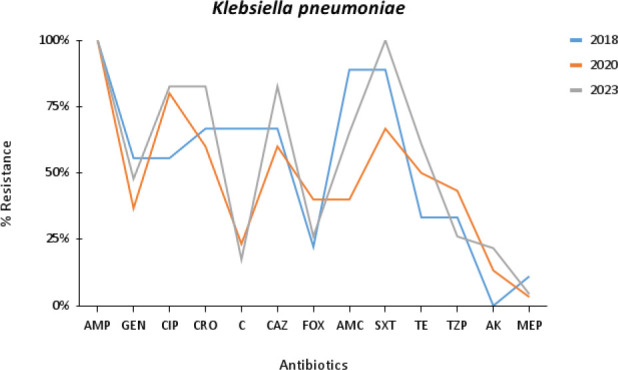
Trends in antimicrobial resistance of *Klebsiella pneumoniae* in 2018, 2020, and 2023. AMP =Ampicillin, GEN= Gentamicin, CIP= ciprofloxacin, TZP= piperacillin-tazobactam, MEP= meropenem, CAZ = ceftazidime, CRO=ceftriaxone, SXT= trimethoprim-sulfamethoxazole, AK= amikacin, TE= tetracycline, C = Chloramphenicol, FOX=cefoxitin, AMC= amoxicillin clavulanic acid. *Klebsiella pneumonia* showed statistically significant difference in resistance to SXT, p=0.02, AMC, p=0.02 and C, p=0.015.

*Acinetobacter spp*. showed high resistance to trimethoprim-sulfamethoxazole, reaching 13 (76.5%) in 2023 compared to 4 (57.1%) in 2018. Resistance to ciprofloxacin also showed a significant change, with rates of 2 (28.6%) in 2018 and 13 (76.5%) in 2023 (p=0.022). Resistance to ceftriaxone varied from high to 16 (94.1%) in 2023, compared to 4 (57.1%) in 2018. Additionally, ceftazidime resistance was significantly different, at a rate of 3 (42.9%) in 2018 and 14 (82.4%) in 2023 (p=0.023). Gentamicin resistance was high at 10 (58.8%) in 2023, compared to 2 (28.6%) in 2018. Resistance to piperacillin-tazobactam was low at 3 (42.9%) in 2018 but high to 13 (76.5%) in 2023. Notably, resistance to meropenem was high at 7 (41.2%) in 2023 compared to 0 (0%) in 2018 (Figure 2C).

**Figure 2C:**
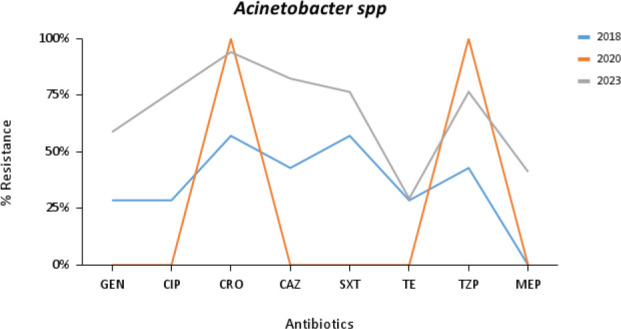
Trends in antimicrobial resistance of *Acinetobacter spp*. in 2018, 2020, and 2023. GEN= Gentamicin, CIP= ciprofloxacin, TZP=piperacillin-tazobactam, MEP= meropenem, CAZ = ceftazidime, CRO=ceftriaxone, SXT= trimethoprim-sulfamethoxazole, TE= tetracycline. Acinetobacter spp. showed statistically significant differences in CIP (p = 0.022) and CAZ (p = 0.023) resistance.

*Pseudomonas spp*. displayed significant variation in ciprofloxacin resistance, with rates of 4 (23.5%) in 2018, 11 (68.8%) in 2020, and 6 (30%) in 2023 (p=0.016). Ceftazidime resistance remains low, with rates of 8 (47.1%) in 2018, 3 (18.8%) in 2020, and 7 (35%) in 2023. Gentamicin resistance was also low, recorded at 4 (23.5%) in 2018, 2 (12.5%) in 2020 and 3 (15%) in 2023. Resistance to Piperacillin-tazobactam varied, with low rates of 5 (29.4%) in 2018 and 6 (30%) in 2023 and high of 9 (56.3%) in 2020. Meropenem resistance remained low throughout the years, with rates of 5 (29.4%) in 2018, 3 (18.8%) in 2020 and 3(15%) in 2023 (Figure 2D).

**Figure 2D:**
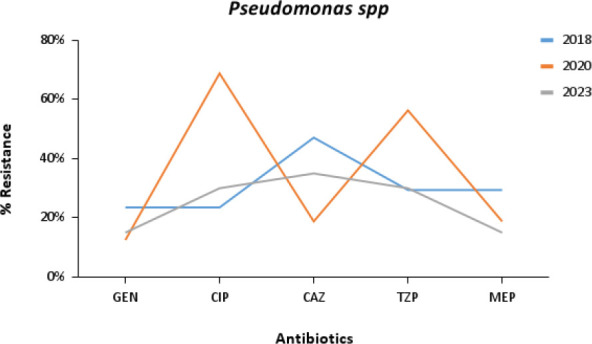
Trends in antimicrobial resistance of *Pseudomonas spp*. in 2018, 2020, and 2023. GEN= Gentamicin, CIP= ciprofloxacin, TZP= piperacillin-tazobactam, MEP= meropenem, CAZ = ceftazidime, *Pseudomonas spp*. showed statistically difference in resistance to CIP, p=0.016.

### Phenotypic determination of ESBL producing organisms

In total, 233 bacterial isolates were tested for ESBL production. Notably, 101(43.3%) isolates showed the ESBL-producing phenotype.

Among the tested strains, the highest frequency of ESBL producing bacteria was observed in *Klebsiella pneumoniae* 40 (64.5%) followed closely by *Escherichia coli* 54 (58.7%). *Acinetobacter spp*. and *Pseudomonas spp*. demonstrated lower frequencies of 2 (7.7%) and 5 (9.4%), respectively. A comparative analysis before and during the COVID-19 era provided interesting insights. Prior to COVID-19, *Klebsiella pneumoniae* displayed ESBL production in 6 (66.7%) cases, while during COVID-19 this percentage slightly decreased to 34 (64.2%). In contrast*, Escherichia coli* showed an increase in ESBL production from 16 (53.3%) before COVID-19 to 38 (61.3%) during COVID-19. In the case of *Acinetobacter spp*., ESBL production decreased from 1 (14.3%) before COVID-19 to 1 (5.3%) during COVID-19. Similarly, ESBL production in *Pseudomonas spp*. decreased from 3 (17.7%) before COVID-19 to 2 (5.6%) during COVID-19 (Table 3).

**Table 3:** Prevalence of ESBL producing bacteria before and During COVID-19 Era

	ESBL Producing	Non ESBL	ESBL Producing	Non ESBL Producing	
Organisms	Before COVID 19		During COVID 19		Total
	N (%)	N (%)	N (%)	N (%)	
*Escherichia coli*	16(53.3)	14(46.7)	38(61.3)	24(38.7)	92
*Klebsiella pneumoniae*	6(66.7)	3(33.3)	34(64.2)	19(35.9)	62
*Pseudomonas spp.*	3(17.7)	14(82.4)	2(5.6)	34(94.44)	53
*Acinetobacter spp.*	1(14.3)	6(85.7)	1(5.3)	18(94.7)	26

Total	26	37	75	95	233

## Discussion

This study investigated the impact of the COVID-19 pandemic on antibiotic resistance in a zonal referral hospital. A key finding was an increase in resistance to several antibiotics during the COVID-19 period including ampicillin, trimethoprim-sulfamethoxazole, amoxicillin-clavulanic acid, ceftriaxone, ceftazidime, ciprofloxacin, tetracycline and piperacillin-tazobactam. Remarkably, the resistance to ampicillin, ciprofloxacin, and ceftriaxone significantly increased. This increase may be attributed to the overuse of these antibiotics as first-line treatments. Supporting this, a recent study in Tanzania found frequent prescriptions of third-generation cephalosporins, amoxicillin-clavulanic acid, ciprofloxacin, gentamicin, and amoxicillin/ampicillin (Katyali *et al.*, 2023). Additionally, increased antibiotic use, particularly beta-lactams and trimethoprim-sulfamethoxazole, has been noted during the COVID-19 pandemic in Tanzania (Sangeda *et al.*, 2024). The global rise in antibiotic resistance during the pandemic has been attributed to increased and often inappropriate antibiotic use, altered prescribing patterns, and healthcare disruptions (Ansari *et al*. 2021). Similar high resistance rates have been observed in other studies (Kishk *et al.*, 2023).

In contrast, the rate of resistance to amoxicillin-clavulanic acid and chloramphenicol decreased. This decrease might be due to changes in treatment protocols during COVID-19, with broad-spectrum antibiotics such as third-generation cephalosporins, azithromycin, tazobactam, fluoroquinolones, and meropenem were mostly used (Malik and Mundra, 2023). Therefore, the decreased use of chloramphenicol and amoxicillin-clavulanic acid reduced the selective pressure on the bacteria to develop resistance. Furthermore, chloramphenicol is prioritized in antibiotic stewardship programs reserved for meningitis (URT-MoHCDGEC 2021). However, these findings differ from other regions. For instance, Taleb *et al*. (2023) reported an increase in resistance to amoxicillin-clavulanic acid in Gaza. Similarly, Aldhwaihi *et al*. (2021) found no change in resistance rates in Riyadh, Saudi Arabia; and noted an increase in chloramphenicol resistance from 0% to 20% during COVID-19. These discrepancies could be due to geographical differences, variations in antibiotic stewardship practices, and the small sample size in our study.

This study found that amikacin and meropenem had lower resistance rates during both periods. This can be explained by their restricted use in severe infections when there are no available alternatives. Controlled use is likely to preserve their effectiveness against critical infections (URT-MoHCDGEC 2021). These findings are consistent with reports of low resistance to amikacin and meropenem (Taleb *et al.*, 2023).

Antibiotic resistance patterns in bacterial isolates before and during COVID-19 have shown alarming trends. Studies have reported increasing resistance in gram-negative bacteria, particularly *Escherichia coli*, *Acinetobacter spp*., *Klebsiella pneumoniae*, and *Pseudomonas spp*., during COVID-19 (Sulayyim *et al.*, 2022). *Escherichia coli* showed the highest resistance to trimethoprim-sulfamethoxazole, ceftriaxone, ceftazidime, ampicillin, tetracycline, amoxicillin-clavulanic acid and ciprofloxacin with statistically significant changes in resistance to ceftriaxone in 2018, 2020 and 2023. *Klebsiella pneumoniae* also showed high resistance to these antibiotics with a significant decrease in resistance to chloramphenicol and amoxicillin-clavulanic acid and a significant change in resistance to trimethoprim-sulfamethoxazole, chloramphenicol and amoxicillin-clavulanic in 2018, 2020 and 2023. This finding alarming public health concerns the challenge of treating diseases caused by these bacterial isolates if the resistance remains high across key antibiotics. Similar trends were reported for increased resistance to ceftazidime, ciprofloxacin, ampicillin and ceftriaxone in both *Escherichia coli* and *Klebsiella pneumoniae* (Khoshbakht *et al.*, 2022; Abdelaziz *et al.*, 2024). However, Despotovic *et al*. (2021) did not observe changes in resistance patterns during COVID-19, and Meena *et al*. (2023) reported decreased resistance to ampicillin, ceftazidime, and tetracycline. These variations may have resulted from geographic variations in bacterial resistance patterns owing to differences in antibiotic usage, healthcare practices, and bacterial genetics (Muteeb *et al.*, 2023).

Furthermore, *Klebsiella pneumoniae* exhibits increasing resistance to piperacillin-tazobactam, amikacin, and cefoxitin. While *Escherichia coli* demonstrates increasing resistance to piperacillin-tazobactam and decreasing resistance rates to aminoglycosides and cefoxitin. These bacteria show the alarming trend of increasing difficult to treat due to resistance to multiple class of antibiotics which limit the available therapeutic option. A similar study reported an increased resistance rate to multiple classes of antibiotics in *Escherichia coli and Klebsiella pneumoniae* (Kitaba *et al.*, 2024).

The findings for *Acinetobacter spp*. showed that resistance rates against ceftriaxone, piperacillin-tazobactam, trimethoprim-sulfamethoxazole, ciprofloxacin, gentamicin, and ceftazidime increased during the pandemic but were not statistically significant, except for ceftriaxone. This result suggests that while overall resistance shows an increase in antibiotic resistance, variation in sample size could be the reason for the lack of statistical significance. When comparing the resistance of *Acinetobacter spp*. by years of isolation, resistance to ciprofloxacin and ceftazidime showed a statistically significant change, resistance was low in 2018 and high in 2023. The study suggests that ciprofloxacin and ceftazidime served as key treatment options with increasing resistance to this antibiotic, reflecting bacterial adaptation and increasing selective pressure due to its overuse. On top of that, our result revealed a worrying trend of high resistance in *Acinetobacter spp*., to gentamicin reached 58.8% in 2023. Aminoglycosides are class of potent antibacterial drugs known for their rapid killing action against bacteria. These antibiotics often maintain their effectiveness in combating resistant infections caused by gram-negative bacteria (Thy *et al.*, 2023). This could be a sign of growing support for gentamicin as a first-line therapy in the case of resistance to beta-lactams and other antibiotic classes, indicating the need for careful monitoring of aminoglycoside use. Our results are consistent with those of other studies that reported high resistance rates of *Acinetobacter spp*. to ceftazidime, ciprofloxacin, piperacillin-tazobactam and aminoglycosides (Abdelmoneim *et al.*, 2024; Golli *et al.*, 2024).

For *Pseudomonas spp*., there was an increase in the resistance to piperacillin-tazobactam and ciprofloxacin during COVID-19. Resistance to ciprofloxacin was significantly higher in 2020 than in 2018 and 2023, likely because of the increased use of these antibiotics, which worsened with increased prescriptions during the pandemic. The lower resistance rate in 2023 may be due to improved antibiotic stewardship. Kishk *et al*. (2023) reported similar findings regarding increased ciprofloxacin resistance during COVID-19. Conversely, Saini *et al*. (2021) reported a decrease in piperacillin-tazobactam resistance but no change in ciprofloxacin resistance during COVID-19. Our study also found decreased resistance to gentamicin and ceftazidime, possibly because of better antimicrobial stewardship. This contrasts with other studies that reported increased resistance to gentamicin and third-generation cephalosporins (Saini *et al*. 2021; Kishk *et al*. 2023). However, Saini *et al*. (2021) observed a decrease in ceftazidime resistance, which is consistent with our findings. Variations in resistance patterns could be due to differences in hospital practices including antimicrobial stewardship and infection control measures.

Carbapenems were considered the last resort for severe gram-negative infections that showed overall low resistance in our study. However, resistance to meropenem in *Acinetobacter spp*. is alarmingly high, from 0% in 2018 to 41.2% in 2023, demanding effective infection control, monitoring, and strengthening antibiotic stewardship. This trend reflects the global increase in carbapenem resistance. For instance, Mirzaei *et al*. (2020) reported 95% resistance in Northern Iran, Shi and Xie (2023) found a 75% resistance rate in China, Tseng *et al*. (2024) reported 65% resistance in Taiwan. These studies confirm our findings of rising global carbapenem resistance in *Acinetobacter spp*., highlighting the limited treatment options for these infections.

Our study revealed the presence of ESBL-producing strains. ESBL production was observed in 43.3% of the bacterial isolates, and *Klebsiella pneumoniae* and *Escherichia coli* were the most common ESBL-producing bacteria. During both study periods, a high prevalence of ESBL production was noted in *Klebsiella pneumoniae* and *Escherichia coli*. This suggested the persistence of these bacteria and their potential contribution to antimicrobial resistance. *Klebsiella pneumoniae* and *Escherichia coli* were dominant ESBL producers aligning with earlier report (El Aila *et al.*, 2023). However, there is a discrepancy concerning *Acinetobacter baumannii*, where El Aila *et al*. (2023) reported a high ESBL production rate of 57.1%. Our results are consistent with those of other studies, indicating a relatively low prevalence of ESBL-producing *A. baumannii* (Namiganda *et al.*, 2019). During the COVID-19 period, the ESBL production rate in *Klebsiella pneumoniae* slightly decreased from 6 (66.7%) before COVID-19 to 34 (64.2%) during the pandemic. In *Escherichia coli*, it increased from 16 (53.3%) before COVID-19 to 38 (61.3%) during the COVID-19 era. These results highlight the differential impact of COVID-19 on antibiotic resistance profiles among bacterial species, which emphasizes the need for improved surveillance, particularly for these bacteria. Similar trends of decreased ESBL-producing *Klebsiella pneumoniae* and increased ESBL-producing *Escherichia coli* have been reported (Abubakar *et al.*, 2023). In contrast, some studies have reported a reduction in ESBL-producing *Escherichia coli* (Hamisi and Yilmaz 2021) and an increase in ESBL-producing *Klebsiella pneumoniae* (Ngoula *et al.*, 2023). This discrepancy may be due to variations in antibiotic prescription patterns, antimicrobial stewardship, and infection control measures during the COVID-19 pandemic.

## Conclusion

Our study revealed significant findings regarding the impact of the COVID-19 pandemic on AMR, offering insights for effective interventions. High AMR rates for certain antibiotics were observed during both periods. The increased use of third-generation cephalosporins, ampicillin, and ciprofloxacin in hospitals during COVID-19 may have contributed to the high AMR rates. Hospital pressures have led to empirical treatments and limited microbiological testing, raising concerns about antibiotic overuse and misuse, and further driving AMR. Restrictive antibiotics, such as Meropenem and Amikacin, showed lower resistance rates and were reserved for severe infections when no alternatives were available. Similarly, chloramphenicol is used for severe infections, such as meningitis, when no alternatives are available. Strengthening antimicrobial stewardship, infection control, and monitoring is crucial for preventing further increases in AMR.

### Conflict of interest statement

The authors declare that there is no conflicts of interest associated with this study.

**List of Abbreviations:**
AMR: Antimicrobial resistance;
AST: Antimicrobial susceptibility test, COVID-19: Coronavirus disease 2019;
DDS: double disk synergy;
ESBL: Extended-spectrum beta-lactamase;
KCMC: Kilimanjaro Christian Medical Center;
KCRI: Kilimanjaro Clinical Research Institute;
WHO: World health organization;
WGS: Whole Genome Sequencing
